# Prognosis of colorectal cancer in Tikur Anbessa Specialized Hospital, the only oncology center in Ethiopia

**DOI:** 10.1371/journal.pone.0246424

**Published:** 2021-02-02

**Authors:** Eyob Kebede Etissa, Mathewos Assefa, Birhanu Teshome Ayele

**Affiliations:** 1 GAMBY College of Medical and Business Sciences, Addis Ababa, Ethiopia; 2 Oncology Department, School of Medicine, Addis Ababa University, Addis Ababa, Ethiopia; 3 Faculty of Medicine and Health Sciences, Division of Epidemiology and Biostatistics, Stellenbosch University, Stellenbosch, South Africa; National Health Research Institutes, TAIWAN

## Abstract

**Introduction:**

Colorectal cancer is the third most commonly diagnosed cancer in males and the second in females worldwide. According to the Addis Ababa cancer registry, it is the first in male and fourth in female in Ethiopia. However, there have not been studies on prognostic factors and survival of colorectal cancer. Hence, this study aimed to estimate survival time and identify prognostic factors.

**Methods:**

In this institution based retrospective study, medical records review of 422 colorectal cancer patients and telephone interview was used as sources of data. Survival time was estimated using Kaplan-Meier estimator. Prognostic factors were identified using the multivariable Cox regression model.

**Results:**

Patients diagnosed with rectal cancer had 76% (HR: 1.761, 95% CI: 1.173–2.644) increased risk of dying compared to colon cancer patients. Node positive patients were 3.146 (95% CI: 1.626–6.078) times likely to die compared to node-negative and metastatic cancer were 4.221 (95% CI: 2.788–6.392) times likely to die compared to non-metastatic patients. Receiving adjuvant therapy reduced the risk of death by 36.1% (HR: 0.639 (95% CI: 0.418–0.977)) compared to patients who had an only surgical resection. The median survival time was 39 months and the overall five years survival rate was 33%.

**Conclusions:**

The overall survival rate was low and a majority of the patients were young at presentation. Patient’s survival is largely influenced by the advanced cancer stage at presentation and delays in the administration of adjuvant therapy. Receiving adjuvant therapy was among the good prognostic factors.

## Introduction

Cancer is the leading cause of death globally, surpassing mortality rates of tuberculosis, malaria and HIV/AIDS combined, and it is quietly taking centre stage. Colorectal cancer is a cancer of the large intestine. Anatomically, it also is known as colon cancer or rectal cancer but when both present with similar features they are termed as colorectal cancer (CRC) [1–5]. Clinical presentation of CRC depends on its size, presence or absence of metastases and tumour location. Early CRC often has no symptoms [6]. CRC is the third most commonly diagnosed cancer in males and second in females, making the disease the second-most common cause of cancer-related death worldwide and remains as one of the biggest killer cancers in the world. Its burden is expected to increase. Despite this increasing burden, in Africa CRC continues to receive a relatively low public health priority due to limited resources and more other prompt diseases [3,5,7,8].

In Ethiopia, CRC is the third most prevalent cancers among the entire adult population and patients often present with advanced stages of cancer [9]. According to the Addis Ababa cancer registry, it is the first in males and fourth in females [10]. From 14,500 cancer-related deaths among males, 11.2% were due to CRC and 4.8% of 26,200 cancer-related deaths in women were due to CRC [11]. Currently, CRC is the most common cancer representing 13% of all malignant tumours in the gastrointestinal tract. More CRC deaths (52%) occur in the less developed regions of the world, reflecting poorer survival in these regions [3,12].

Various studies have shown that CRC survival is mainly determined by age at diagnosis, complicated colorectal cancer (perforation, obstruction, bleeding), tumour site, tumour grade, tumour maturity, tumour size, lymph node involvement, distant metastasis, treatment modalities and complication after treatment. Some literature reported that sex, educational status, family history, pre-operative carcinoembryonic Antigen (CEA) level and lymphovascular invasion as significant prognostic factors [13–16]. The highest five-year survival rates (72%) were in Israel and North Korea, in North America, Europe and Australia/New Zealand survival varies from 65%-70%, 55% in other developed countries, 39% in developing countries including India and 14% in Africa [6,17,18].

In Africa, data for CRC survival is scanty. In Ethiopia, to the best of our knowledge, there is no study on prognostic factor and survival of colorectal cancer. For these reasons, this study explored the survival and prognostic factors of colorectal cancer patients in Tikur Anbessa Specialized Hospital (TASH) of Addis Ababa, Ethiopia.

## Materials and methods

A retrospective study was conducted on colorectal cancer patients referred to TASH Radiotherapy centre from January 2012 to December 2016. TASH is the only oncology and cancer referral centre providing services for the majority of cancer cases from all over the country [19].

The medical records of 422 colorectal cancer patients were reviewed and telephone follow-up was used to find out the vital status of patients. The follow-up time was from the first date of confirmed diagnosis to the date of death, date of loss to follow up or date of the last contact. Patients with unknown vital status or alive patients during last contact (phone) were considered as censored. Thirteen patients not meeting the inclusion criteria were excluded from the study. Data on socio-demographic characteristics, genetic factor (family history of CRC), comorbidities (HIV/AIDS, diabetic mellitus and hypertension), and pathological characteristics (primary tumour site, histopathology, tumour grade, size of the tumour, lymph node metastasis, distant metastasis, lymphovascular invasion, perineural invasion and pre-operative CEA level and treatment modality) were extracted from the medical records.

### Sample size consideration

Sample size determination started by determining d (number of events) because of the number of observed events matters beyond the total number of patients in survival data analysis. The required total number of events (Freedman 1982) was calculated using [20]:

d = (Zα2+Zβ)24InΔ2, assuming that *Δ* = hazard ratio is constant in time. For the two-sided log-rank test (H_0_: *Δ =* 1 *vs* H_A_: *Δ* ≠ 1).

Then, the required total number of patients can be calculated as $\frac{d}{Probablity\;(of\;an\;event)}$. A previous similar study conducted in Ghana showed that survival function (s (t)) = 0.16, thus we used *s*(*t*) = 0.16. We also know that Δ = −log(*s*(*t*)) = log(0.16) = 1.832.

To obtain the total sample size required to yield a power of 90%, a hazard ratio of 1.832 and 5% two-sided level of significance, the required number of events is:

d=(Zα2+Zβ)24InΔ2=(1.96+1.282)24In(1.832)2=115.

Z_α/2_ = 1.96 for 95% confidence and Z_β =_ probability of committing type II error to give power of 90%.

Accordingly, $n=\frac{d}{Probablity\;(of\;an\;event)}=\frac{115}{0.84}=137$.

Adjusting for a loss of 20%. $n_{adj}=\frac{n}{1-loss}=\frac{137}{1-0.2}\approx 172$.

The required sample size was 172. However, all colorectal cancer patients treated in TASH from 2012 to 2016 and who fulfil the inclusion criteria were included in the study. Hence the sample used for this study was 422.

### Analysis plan

The analysis was performed using SPSS version 25 and Stata version 14. Descriptive analysis was done using frequency, percentages and median. Survival time was estimated using the Kaplan-Meier method. A log-rank test was used to compare (overall) survival of two or more groups and identify potential prognostic variables. In the univariate analysis, variables with p < 0.25 were entered into the multivariable Cox regression model. Deviation from the proportional hazard assumption of the Cox regression model was examined using plots of scaled Schoenfeld residuals, global test and log-log transformation of Kaplan- Maier survival curves of categorical variables. Prognostic factors of colorectal cancer survival were identified using multivariable Cox regression model. P-value of less than 0.05 was considered as statistically significant.

### Ethical approval and consent to participate

To access the medical records ethical approval letter for the proposed study was obtained from GAMBY Medical and Business Sciences College institutional research ethics review committee (GCA.A 20/2010) to Tikur Anbessa Specialized Hospital. Permission letter was obtained from Tikur Anbessa Specialized Hospital to extract data from medical records. Written consent could not be obtained as the patients were not at the facility. Verbal informed consent was obtained for patient’s eligible for the phone interview, witnessed by two experienced data collectors who are not a part of the study and documented via written note. Minors were not included in the study.

## Results

### Summary of baseline information

A total of 422 colorectal cancer patients treated in Tikur Anbessa Specialized Hospital from January 2012 to December 2016 were considered in this retrospective study. One hundred seventy-five (41.5%) of the patients died in the study period while the remaining 247 (58.5%) were censored. Majority of the patients, 262 (62.1%) were male. The overall median age was 46 years with Interquartile range (IQR) of 23 and 185 (43.9%) were in the age group of 40–59 years. Fifty-two (12.3%) patients comorbidities 28 (6.6%) of the patients were hypertensive, 15 (3.6%) were diabetic and 7(1.7%) were HIV positive at presentation. The details are presented in Table 1.

**Table 1:** Socio-demographic characteristics and co-morbidity history of colorectal cancer patients (n = 422) in Tikur Anbesa Specialized Hospital (TASH), Addis Ababa, Ethiopia, 2012–2016.

Variables	n (%)
**Age** (Median, IQR) 46 (23)	
≤ 29	50 (11.8)
30–39	79 (18.7)
40–49	94 (22.3)
50–59	91 (21.6)
60–69	77 (18.2)
≥70	31 (7.3)
**Sex**	
Male	262 (62.1)
Female	160 (37.9)
**Place of residence**	
Addis Ababa	187 (44.3)
Out of Addis Ababa	235 (55.7)
**Educational status**	
Unable to read and write	16 (3.8)
Able to read and write	2 (0.5)
Elementary (1–8)	20 (4.7)
High school (9–12)	19 (4.5)
College and above	22 (5.2)
Unspecified	343 (81.3)
**Family history of CRC**	
Yes	1 (0.2)
No	132 (31.3)
Unspecified	289 (68.5)
**Comorbidities**	
Yes	52 (12.3)
No	370 (87.7)
**HIV/AIDS**	
Yes	7 (1.7)
No	415 (98.3)
**Diabetic mellitus**	
Yes	15 (3.6)
No	406 (96.2)
Unspecified	1 (0.2)
**Hypertension**	
Yes	28 (6.6)
No	394 (93.4)

IQR = inter-quartile range, HIV/AIDS = human immune virus/acquired immune deficiency syndrome.

As depicted in Table 2, colon, 217 (51.4%), and rectum, 144 (34.1%), were the common cancer sites involved. Forty-five (31.3%) rectal cancer patients had adjuvant chemotherapy or radiotherapy after surgery, 31 (21.5) had adjuvant chemotherapy and radiotherapy after surgery and 23 (16%) did not receive any treatment. Majority of the patients, 333 (∼79%), have adenocarcinoma NOS, followed by mucinous adenocarcinoma (12.9%) and signet ring adenocarcinoma (4.7%). The majority of patients, 198 (46.9%), had well-differentiated adenocarcinoma.

**Table 2:** Pathological and clinical characteristics of colorectal cancer patients (n = 422) in TASH, Addis Ababa, Ethiopia, 2012–2016.

Variables		n (%)
**Primary tumour site**		
Colon		217 (51.4)
Rectum		144 (34.1)
Treatment modalities for rectal cancer	Surgery only	27 (18.7)
	Chemotherapy OR radiotherapy only	14 (9.7)
	Surgery + chemotherapy OR radiotherapy	45 (31.3)
	Surgery + chemotherapy + radiotherapy	31 (21.5)
	Chemotherapy + radiotherapy	4 (2.7)
	None	23 (16)
Colorectal		31 (7.3)
Recto sigmoid		30 (7.1)
**Histopathology**		
Adenocarcinoma NOS		333 (78.9)
Mucinous adenocarcinoma		53 (12.6)
Signet ring cell adenocarcinoma		20 (4.7)
Tubulovillous adenocarcinoma		7 (1.7)
Other		5 (1.2)
Unspecified		4 (0.9)
**Tumour grade**		
Well differentiated		198 (46.9)
Moderately differentiated		84 (19.9)
Poorly differentiated		30 (7.1)
Undifferentiated		4 (0.9)
Unspecified		106 (25.1)
**Size of the tumour**		
TX		12 (2.8)
T1		7 (1.7)
T2		62 (14.7)
T3		197 (46.7)
T4		113 (26.8)
Unspecified		31 (7.3)
**Lymph node metastasis**		
Node negative		79 (18.7)
Node positive		102 (24.2)
Node unknown		241 (57.1)
**Distant metastasis**		
MX		37 (8.8)
MO		230 (54.5)
M1		143 (33.9)
Unspecified		12 (2.8)
**Lymph vascular invasion**		
Yes		6 (1.4)
No		38 (9.0)
Unspecified		378 (89.6)
**Perineural invasion**		
Yes		2(0.5)
No		2 (0.5)
Unspecified		418 (99.1)
**Pre-operative CEA level**		
Not elevated (≤5ng/ml)		16 (3.8)
Elevated (>5ng/ml)		14 (3.3)
Unspecified		392 (92.9)
**Treatment modalities**		
Surgery only		98 (23.2)
Chemotherapy OR radiotherapy only		25 (5.9)
Surgery + chemotherapy OR radiotherapy		208 (49.3)
Surgery + chemotherapy + radiotherapy		49 (11.6)
Chemotherapy + radiotherapy		6 (1.4)
None		36 (8.5)

NOS = not otherwise specified, CEA = carcinoembryonic Antigen.

Majority of the patients were diagnosed with a primary tumour of T3 (46.7%). About one-fourth patients presented with pathologically confirmed regional lymph node involvement. In 57.1% of cases, the lymph node status is unknown which means we do not know the TNM staging in this group of patients. Nearly half, (49.3%), of the patients, had received adjuvant chemotherapy or radiotherapy after surgery. All patients who underwent adjuvant therapy had surgery.

### The yearly incidence of death and survival of colorectal cancer patients

Among the total of 422 patients, 175 (41.5%) died and 144 (34.1%) were alive by October 2018, when patients status was last updated. The vital status of the remaining, 103 (24.4%), was unknown due to refusal to respond to calls and no working telephones. Overall, there was 822.25 person-years observation with a median follow-up time of 19 months (IQR 28). This makes the incidence rate of death of colorectal cancer 21.28% per 100 person-years observation.

**Fig 1:**
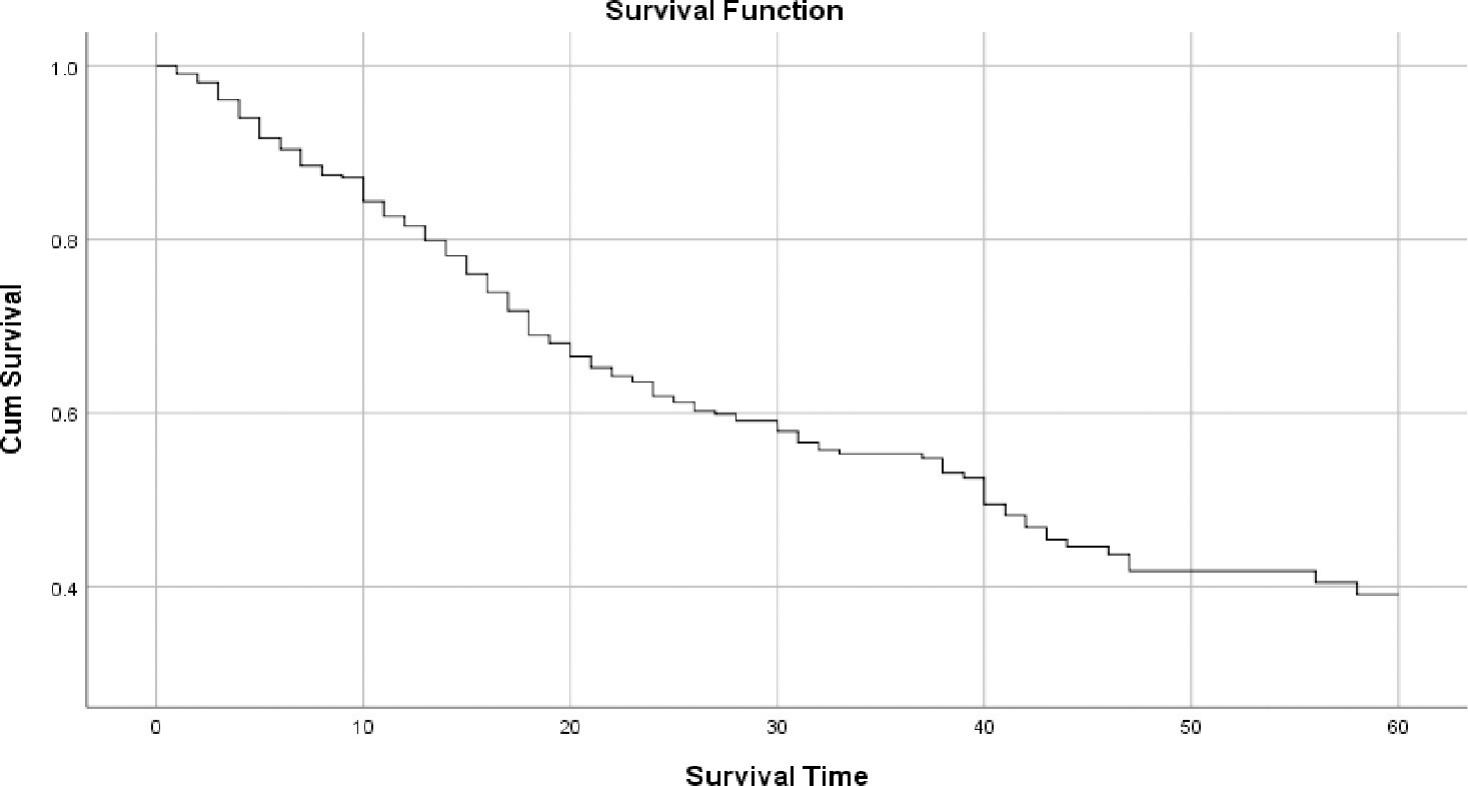
Overall survival probability of colorectal cancer patients in TASH, Addis Ababa, Ethiopia, 2012–2016.

The graph in Fig 1 shows the overall survival curve of colorectal cancer patients in TASH. The graph shows that patients’ survival decreases as the observation time increases. One, three and five years overall survival rate were 80%, 55%, and 33% respectively. The median overall survival time was 39 months.

Table 3 shows the results of the Log-rank test of socio-demographic characteristics and the presence of comorbidities in CRC patients. No statistically significant differences were observed among the survival groups (P>0.05) although having comorbidities seemed to lower patients’ survival rate.

**Table 3:** Median survival, survival rate and log-rank test for univariate analysis of socio-demographic characteristics and comorbidities.

Variable	n	Median survival in month	Five-year survival rate (%)	Log-rank test		
				*x*^2^(df)		P-value
**Age**				7.96(5)	0.159	
≤29	50	23	29			
30–39	79	39	45			
40–49	94	43	35			
50–59	91	40	32			
60–69	77	60	50			
≥70	31	25	7			
**Sex**				5.82(1)	0.468	
Male	262	39	33			
Female	160	46	34			
**Place of residence**				0.26(1)	0.871	
Addis Ababa	187	39	36			
Out of Addis Ababa	235	40	29			
**Comorbidities**				2.70(1)	0.100	
Yes	52	21	23			
No	370	41	35			
**HIV/AIDS**				2.09(1)	0.148	
Yes	7	13	29			
No	415	40	33			
**Diabetic mellitus**				0.74(2)	0.964	
Yes	15	60	26			
No	406	40	35			
**Hypertension**				0.50(1)	0.477	
Yes	28	60	60			
No	394	39	31			

**x**^**2**^ **= chi square**, DF = degree of freedom, HIV/AIDS = human immune virus/acquired immunodeficiency syndrome.

The median survival time, survival rate and log-rank test for pathological and clinical variables were assessed univariately.

As shown in Table 4, pathological and clinical factors like primary tumour site, histopathology, tumour grade, size of the tumour, lymph node metastasis, distant metastasis and treatment modality were significantly associated with patients’ survival time (p<0.05). Colon and colorectal cancer patients had better survival compared to that of the other commonest cancer site rectum. Patients with poorly differentiated tumour grade were at increased risk of death compared to those with well and moderately differentiated tumour grades. The median survival time was higher (60 months) among those node-negative patients compared to those diagnosed with node-positive (24 months). Similarly, metastatic cancer patients had lower 5-year survival rate (2%). According to treatment modality, patients who received adjuvant chemotherapy and adjuvant radiotherapy after surgery and who received adjuvant chemotherapy or adjuvant radiotherapy after surgery had higher median survival time (73 months) and (46 months) respectively. Lower median survival time (6 months) was observed among those who did not receive any cancer treatment during the follow-up time in TASH. This is because patients with advanced disease are not fit for chemotherapy or radiotherapy and refusal to initiate treatment. Enough data was not obtained for variables like pre-operative CEA level, lymphovascular and perineural invasion. Hence, not data exploration was performed for these variables.

**Table 4:** Median survival, survival rate and log-rank test for univariate analysis of pathological and clinical characteristics.

Variable	n	Median survival in month	Five-year survival rate (%)	Log-rank test	
				*x*^2^(df)	P-value
**Primary tumour site**				17.00(3)	**0.001**
Colon	217	60	53		
Rectum	144	25	16		
Colorectal	31	57	21		
Recto sigmoid	30	41	27		
**Histopathology**				14.93(5)	**0.011**
Adenocarcinoma NOS	333	42	36		
Mucinous adenocarcinoma	53	24	15		
Signet ring cell adenocarcinoma	20	23	-		
Tubulovillous adenocarcinoma	7	5	-		
**Tumour grade**				52.63(4)	**0.0001**
Well differentiated	198	60	57		
Moderately differentiated	84	41	31		
Poorly differentiated	30	15	41		
Undifferentiated	4	40	-		
Unspecified	106	18	6		
**Size of the tumour**				55.46(5)	**0.0001**
TX	12	12	-		
T1	7	52	-		
T2	62	60	68		
T3	197	46	38		
T4	113	21	22		
Unspecified	31	15	-		
**Lymph node metastasis**				34.584(2)	**0.0001**
Node negative	79	60	77		
Node positive	102	24	14		
Node unknown	241	32	28		
**Distant metastasis**				142.60(3)	**0.0001**
MX	37	30	17		
M0	230	60	55		
M1	143	14	2		
**Treatment modalities**				66.09(5)	**0.0001**
Surgery only	98	39	25		
Surgery OR radiotherapy only	25	14	-		
Surgery + chemotherapy OR radiotherapy	208	46	43		
Surgery + chemotherapy + radiotherapy	49	73	38		
Chemotherapy + radiotherapy	6	12	-		
None	36	6	14		

**x**^**2**^ **= chi square**, DF = degree of freedom, NOS = not otherwise specified.

### Result of a multivariable Cox regression model

To identify prognostic factors, variables with p-value < = 0.25 at the univariate analysis (Tables 3 and 4) were considered for the multiple Cox PH regression model. Hence, age at diagnosis (p = 0.159), comorbidity (p = 0.100), primary tumor site (p<0.01), histopathology of CRC (p = 0.011), tumor grade (p<0.001), size of the tumor (p<0.001), lymph node metastasis (p<0.001), distant metastasis (<0.001), treatment modality (p<0.001) and sex (due to its importance in oncology study) were entered to the final multiple Cox regression model. The proportional hazard assumption was tested using scaled-Schoenfeld residuals and the global test for the model (p = 0.0584). We failed to reject the null hypothesis that the hazards are proportional. The scaled Schoenfeld residuals were also plotted for each variable and the slope was closed to zero for variables in the model and Kaplan Meier curves were parallel for each category of variables versus time. These imply that the proportional hazard assumption was not violated for this data. Results of the final multivariable Cox regression analysis are presented in Table 5. From greatest to least powerful: distant metastasis (P <0.001), lymph node involvement (P <0.01), treatment modalities (p< 0.01) and primary tumour site (P <0.05) were significantly associated with survival rate.

**Table 5:** Multivariable survival analysis of prognostic factors in colorectal cancer patients.

Prognostic variables	Estimate	Standard error	p-value	HR	CI (95%)
**Age**			0.263		
≤29				1	
30–39	-0.266	0.329	0.419	0.767	0.403–1.460
40–49	-0.170	0.327	0.582	0.836	0.442–1.582
50–59	-0.067	0.300	0.822	0.935	0.520–1.682
60–69	0.046	0.325	0.908	1.038	0.550–1.958
≥70	-0.740	0.376	0.052	0.484	0.233–1.006
**Sex**					
Male	0.291	0.175	0.097	1.338	0.949–1.887
Female				1	
**Comorbidities**					
Yes	0.146	0.239	0.543	1.157	0.724–1.848
No				1	
**Primary tumour site**			**0.036**		
Colon				1	
Rectum	0.566	0.207	0.006	1.761	1.173–2.644
Colorectal	-0.023	0.355	0.948	0.977	0.488–1.959
Recto sigmoid	0.446	0.339	0.188	1.562	0.804–3.035
**Histopathology**			0.249		
Adenocarcinoma NOS				1	
Mucinous adenocarcinoma	0.373	0.245	0.128	1.452	0.899–2.346
Signet ring cell adenocarcinoma	0.487	0.388	0.210	1.628	0.760–3.485
Tubulovillous adenocarcinoma	0.147	0.522	0.778	1.159	0.417–3.223
**Tumour grade**			1.180		
Well-differentiated				1	
Moderately differentiated	0.311	0.221	0.159	1.364	0.885–2.102
Poorly differentiated	0.579	0.325	0.066	1.817	0.060–3.439
Undifferentiated	1.220	1.055	0.247	3.389	0.429–26.786
**Size of the tumour**			0.051		
T1	0.112	0.628	8.859	1.118	0.326–3.832
T2	-0.336	0.323	0.297	0.714	0.380–1.344
T3	-0.195	0.199	0.326	0.823	0.557–1.216
T4				1	
**Lymph node metastasis**			**0.003**		
Node negative				1	
Node positive	1.146	0.336	0.001	3.146	1.629–6.078
**Distant metastasis**			**0.000**		
M0				1	
M1	1.440	0.212	0.000	4.221	2.788–6.392
**Treatment modalities**			**0.007**		
Surgery only				1	
Surgery OR radiotherapy only	-0.051	0.334	0.879	0.950	0.494–1.828
Surgery + chemotherapy OR radiotherapy	-0.447	0.217	0.039	0.639	0.418–0.977
Surgery + chemotherapy + radiotherapy	-0.653	0.319	0.041	0.521	0.279–0.973
Chemotherapy + radiotherapy	0.142	0.572	0.803	1.153	0.376–3.537
None	0.575	0.350	0.100	1.778	0.895–3.531

HR = hazard ratio, CI = confidence interval, NOS = not otherwise specified.

Patients diagnosed with rectal cancer had 76% (HR 1.761, 95% CI: 1.173–2.644) increased risk to die compared to colon cancer. Node positive patients were 3.146 (95% CI: 1.629–6.078) times likely to die compared to node-negative patients. The risk of death for metastatic cancer was 4.221 (95% CI: 2.788–6.392) times to non-metastatic patients. For treatment modality, risk of death was 36.1% lesser (HR: 0.639 (95% CI: 0.418–0.977)) and 47.9% lesser (HR: 0.521 (95% CI: 0.279–0.973)) in those patients who received adjuvant chemotherapy or adjuvant radiotherapy and who received adjuvant chemotherapy and adjuvant radiotherapy, respectively compared to patients who had only surgical resection (Table 5).

## Discussion

This study provided estimates of survival time and identified prognostic factors of colorectal cancer patients in the only oncology referral Centre in Ethiopia over the period 2012–2016. Our findings revealed that the median age at diagnosis was 46 and 52.8% of those diagnosed were under 50, which shows the majority of colorectal cancer patients in Ethiopia are young adults and middle-aged adults whereas the median age at diagnosis was 70 in Germany. Data from Oman showed that the median age was 56 years and a majority of the patients, 61.1%, were older than 50 years. Similarly, in Jordan, the median age was 62 for males and 58 for females. More than half of the patients were above age 60 years, which differs from our study result. The age structure of Ethiopia could be a contributing factor. It might also relate to the unhealthy nutritional habit in Ethiopia (high intake of fats and red meats) and lack of birth registration may contribute as most patients from the rural area might not know their exact age [21–23].

Around 34% of the patients had metastatic cancer at presentation. This is similar to a report from Oman (36.8) and Kenya (29.1%) but in contrast with reports from Germany (28.1%), in Ghana (24.9%), in Malaysia (24.3%), in Jordan (17.2%) and Iran (6.8%) of patients were metastatic [13,16,21–25]. The late presentation might be due to patients’ low awareness of colorectal cancer sign and symptoms, lack of screening program, lengthy and poor referral system in Ethiopia. This might have contributed to the observed low survival of patients treated in Tikur Anbesa Specialized Hospital in Addis Ababa, Ethiopia.

In our current study, the five-year overall survival (OS) rate was 33%, much lower than the OS rate in developed countries. Highest five-year survival rate (72%) are in Israel and North Korea. Survival varies from 65% to 70% in North America, Europe and Australia, 63% in Germany and 55% in other developed countries [17,18,21]. This could be primarily due to increase in colorectal cancer screening, removal of precancerous adenomas, timely and advanced treatment in developed countries. In developing Asian countries, the five-year survival rate of CRC patients ranged from 33% to 56.9% in Iran, 58.2% in Jordan, 43% & 48.7% in Malaysia, 42% in Oman, 39% in India, 38.6% in Thailand which are above the survival rate of patients in Ethiopia [13–15,18,22–26]. The reason for this difference could be lack of screening programs to detect cancer at an early stage, poorly developed infrastructure of cancer health cares in Ethiopia. Treatments are also very limited and inaccessible especially for patients living far from TASH, the only oncology and cancer referral centre in the country. The five years overall survival rates of colorectal cancer patients in a teaching hospital in Ghana (n = 221) was 16% which is below the overall survival rate in this study [16]. Variation in methodology, population characteristics and observed number of events may have contributed to these differences in findings.

In our study, age at diagnosis was not a significant prognostic factor affecting survival. This is consistent with studies conducted in Malaysia and Ghana [16,25]. However, it was inconsistent with studies conducted in India, Iran, Jordan and Thailand [13,14,23,27,28]. This might be because the majority of the study participant was young compared to patients in those studies. Comorbidity is not also a significant prognostic factor affecting patient’s survival. This finding is also in line with reports from Ghana, Iran and Malaysia [15,16,25].

In multivariable Cox regression model, primary tumor site (P <0.05), lymph node involvement (P <0.01), distant metastasis (P <0.001) and treatment modalities (p< 0.01) were significant prognostic factors for colorectal cancer patients survival. Patients diagnosed with rectal cancer had 76% (HR = 1.761) increased hazard to death compared to colon cancer patients. This is in agreement with reports from Iran where rectal cancer had a high risk of death and determines prognosis significantly [13]. In our study, it is associated with inadequacy of treatment.

In this study, the hazard of death was significantly higher for those patients who were diagnosed with a positive lymph node with (HR = 3.146) compared to node-negative. This finding is in line with reports from Ghana, Malaysia, Thailand and Iran [13,14,16,25]. The other finding of this study is the risk of death was four-fold higher (HR = 4.221) for those who presented with metastatic cancer compared to non-metastatic cancer patients. This finding is comparable to those reports found in Ghana, Thailand, Jordan, Malaysia and Iran [13,14,16,23,25]. Node involvement and metastatic cancer at presentation were the major prognostic factors for colorectal cancer patients.

The finding of this study demonstrates that the risk of death was significantly lesser (HR = 0.639 &0.521) among those who received adjuvant chemotherapy or/and adjuvant radiotherapy compared to those who only received surgical resection. This is similar to reports from Ghana, Thailand, Iran and Malaysia that adjuvant therapy was a good prognostic factor [13,14,16,25].

The study was conducted in the only oncology referral centre in the country that provides services for the majority of cancer cases from all over the country. Hence, our findings may reflect the situation in the whole of Ethiopia. As the data is from a secondary source, some prognostic factors such as family history, lymph node involvement, lymph vascular invasion, perineural invasion and pre-operative CEA level were missing from medical records during data collection and cleansing. Also, important information on diet and physical activity, alcohol intake, smoking history, body mass index and complication after treatment were not included in our study. The other limitation is that only seven CRC patients died in the institution with a report of detailed causes of death. The death of the remaining 168 patients and causes of deaths were ascertained by telephone interview, not from death report which may lead to over detection of death as a result of cause ascertainment bias.

## Conclusion

This study estimates a low overall five-year survival rate. Majority of the patient’s s were young adults and diagnosed with advanced cancer stage. Patient’s survival is largely influenced by advanced stage at presentation and delayed in the administration of adjuvant therapy. Primary tumour site, lymph node involvement, distant metastasis and treatment modalities were important prognostic factors for colorectal cancer. Rectal cancer, node-positive, and metastatic cancer at a presentation in colorectal cancer patients decreased the survival rate. Whereas, adjuvant therapy increased survival rate. Administration of adjuvant chemotherapy through improving access to chemotherapy and radiotherapy will improve outcome.

## Supporting information

S1 Data (SAV)

## References

[1] Boutayeb A. The double burden of communicable and non-communicable diseases in developing countries. Transactions of The Royal Society of Tropical Medicine and Hygiene. 2006;100(3):191–9. 10.1016/j.trstmh.2005.07.021 16274715

[2] Olsen M. Cancer in Sub-Saharan Africa: The Need for New Paradigms in Global Health. The Frederick S Pardee Center for the Study of the Longer-Range Future 2015.

[3] Lindsey A, Bray F, Siegel Rebecca L, Ferlay J, Tieulent Lortet-J, Jemal A. Global Cancer Statistics, 2012 CA CANCER J CLIN. 2015;65(2):87–108. 10.3322/caac.21262 25651787

[4] About Colorectal Cancer. American Cancer Society. 2016.

[5] Bray F, Ferlay J, Soerjomataram I, L. Siegel R, A. Torre L, Jemal A. Global Cancer Statistics 2018: GLOBOCAN Estimates of Incidence and Mortality Worldwide for 36 Cancers in 185 Countries. CA CANCER J CLIN 2018;68:394–424. 10.3322/caac.21492 30207593

[6] American Cancer Society Colorectal Cancer Facts & Figures 2014–2016. 2014.

[7] Global, regional and national levels of age-specific mortality and 240 causes of death, 1990–2013: a systematic analysis for the Global Burden of Disease Study 2013. 1990–2013 The Lancet. 2015:385. 10.1016/S0140-6736(14)61682-2 PMC4340604 25530442

[8] Irabor D. Emergence of colorectal cancer in West Africa: Accepting the inevitable. The South African Gastroenterology Review. 2017(1):11–6. 10.4103/0300-1652.234076 29962648 PMC6009139

[9] National Cancer Control Plan of Ethiopia 2015.

[10] Memirie S, Habtemariam M, Asefa M, Deressa B, Abayneh G, Tsegaye B, et al Estimates of Cancer Incidence in Ethiopia in 2015 Using Population-Based Registry Data. Journal of Global Oncology. 2018 10.1200/JGO.17.00175 30241262 PMC6223441

[11] World Health Organization—Cancer Country Profiles. 2014.

[12] Cancer fact sheets: Colorectal cancer. WHO International Agency for Research on Cancer. 2016.

[13] Zare-Bandamiri M, Khanjani N, Jahani Y, Mohammadianpanah M. Factors Affecting Survival in Patients with Colorectal Cancer in Shiraz, Iran. Asian Pacific Journal of Cancer Prevention. 2016;17(1):159–63. 10.7314/apjcp.2016.17.1.159 26838203

[14] Laohavinij S, Maneechavakajorn J, P T. Prognostic factors for survival in colorectal cancer patients. J Med Assoc Thailand. 2010;93(10):1156–66. 20973318

[15] Rasouli M, Moradi G, Roshani D, Nikkhoo B, Ghaderi E, Ghaytasi B. Prognostic factors and survival of colorectal cancer in Kurdistan province, Iran: A population-based study (2009–2014). Medicine (Baltimore). 2017;96(6). 10.1097/MD.0000000000005941 PMC5312991 28178134

[16] Agyemang-Yeboah F, Yorke J, Obirikorang C, Batu E, Acheampong E, Frimpong E, et al Colorectal cancer survival rates in Ghana: A retrospective hospital-based study. PLOS ONE. 2018 10.1371/journal.pone.0209307 30566456 PMC6300283

[17] Granados-Romero J, Valderrama-Treviño A, Contreras-Flores E, Barrera-Mera B, Enríquez M, Uriarte-Ruíz K, et al Colorectal cancer: a review. International Journal of Research in Medical Sciences. 2017;5(11):4667–76.

[18] Global Cancer Facts & Figures 4th Edition American Cancer Society, Inc, Surveillance Research 2018.

[19] Gebremedhin A, Shimelis D, Aster T, Shad A, M L. International network for cancer treatment and research. 2012.

[20] Freedman LS. Tables of the number of patients required in clinical trials using the log rank test. Statistics in Medicine. 1982;1(2). 10.1002/sim.4780010204 7187087

[21] Majek O, Gondos A, Jansen L, Emrich K, Holleczek B, Katalinic A, et al Survival from colorectal cancer in Germany in the early 21st century. British Journal of Cancer. 2012;106:1875–80. 10.1038/bjc.2012.189 22555397 PMC3364110

[22] Kumar S, Burney I, Zahid K, Souza P, Belushi M, Meki T, et al Colorectal Cancer Patient Characteristics, Treatment and Survival in Oman—a Single Center Study. Asian Pacific Journal of Cancer Prevention. 2015;16(12):4853–8. 10.7314/apjcp.2015.16.12.4853 26163603

[23] Sharkas G, Arqoub K, Khader Y, Tarawneh M, Nimri O, Al-zaghal M, et al Colorectal Cancer in Jordan: Survival Rate and Its Related Factors. Journal of Oncology 2017 10.1155/2017/3180762 28458690 PMC5387838

[24] Saidi H, Abdihakin M, Njihia B, Jumba G, Kiarie G, Githaiga J, et al Clinical Outcomes of Colorectal Cancer in Kenya. The Annals of African Surgery. 2011;7.

[25] Hassan M, Suan M, Soelar S, Mohammed N, Ismail I, Ahmad F. Survival Analysis and Prognostic Factors for Colorectal Cancer Patients in Malaysia. Asian Pacific Journal of Cancer Prevention. 2016;17(7):3575–81. 27510011

[26] Magaji B, Moy F, Roslani A, Law W. Survival rates and predictors of survival among colorectal cancer patients in a Malaysian tertiary hospital. BMC Cancer 2017;17. 10.1186/s12885-017-3336-z PMC5437641 28521746

[27] Yeole BB, Sunny L, Swaminathan R, Sankaranarayanan R, DM P. Population-based survival from colorectal cancer in Mumbai, (Bombay) India. Eur J Cancer. 2001;37(11):1402–8. 10.1016/s0959-8049(01)00108-3 11435072

[28] Asl M, Barfaeie F, Gohari M, Roudbary M, Khodabakhshi R. Survival analysis of colorectal cancer patients and its prognostic factors using Cox regression Razi Journal of Medical Sciences 2015;22(130).

