# Supplementary material for: Prognosis of colorectal cancer in Tikur Anbessa Specialized Hospital, the only oncology center in Ethiopia
Source: PLoS One. 2021 Feb 2;16(2):e0246424. doi: 10.1371/journal.pone.0246424 (PMC7853488; doi:10.1371/journal.pone.0246424)
Supplement: Attachment — Submitted filename: notes to R1 version.docx [file pone.0246424.s003.docx]

PONE-D-20-03951

Prognosis of Colorectal Cancer in Tikur Anbessa Specialized Hospital, the Only Oncology Center in Ethiopia.

**Section Material and Methods**:

The sentence:

„Thirteen patients not meeting the exclusion criteria were excluded from the study.“

Should sound as

„Thirteen patients not meeting the inclusion criteria were excluded from the study.“

**Section: Sample size consideration:**

Citation: Freeman 1982 is not included to the References list

Symbols as: Z_α/2_, Z_β,_  s(t), P…….. are not explained or commented.

**Table 1:**

Family history - of (any) cancer or of only CRC?

**Table 2, Table 4 and Table 5**

Size and location of the tumor

Should be only Size of the tumor (no info about location, relevant data about location are under „Primary tumor site“).

**Section: Result of multivariable Cox regression model**

Unify the style of writing of capital letters in list of variables over the whole section. In this list is again idiom „size and location of tumor“ – should be only „size of tumor“.

**Section: Discussion**

Line 323: unify style of writing capital letters for variables
