# Supplementary material for: Prognosis of colorectal cancer in Tikur Anbessa Specialized Hospital, the only oncology center in Ethiopia
Source: PLoS One. 2021 Feb 2;16(2):e0246424. doi: 10.1371/journal.pone.0246424 (PMC7853488; doi:10.1371/journal.pone.0246424)
Supplement: Attachment — Submitted filename: Responce to reviewers.docx [file pone.0246424.s004.docx]

**Comments from the editor**

**Section Material and Methods**:

The sentence:

„Thirteen patients not meeting the exclusion criteria were excluded from the study.“

Should sound as

„Thirteen patients not meeting the inclusion criteria were excluded from the study.“

**Response:** Thank you. We have amended the text to make a sound.

**Section: Sample size consideration:**

Citation: Freeman 1982 is not included to the References list

Symbols as: Z_α/2_, Z_β,_  s(t), P…….. are not explained or commented.

**Response:** The suggested correction have been made in the specified section. Now it reads:

**Sample size consideration**

Sample size determination started by determining d (number of events) because of the number of observed events matters beyond the total number of patients in survival data analysis. The required total number of events (Freedman 1982) was calculated using:[20]

d =$\left( Z_{\frac{\alpha}{2}}+Z_{\beta} \right)^{2}\frac{4}{{In\Delta}^{2}} ,assuming$that *Δ*=hazard ratio is constant in time. For the two-sided log-rank test (H_0_: *Δ =* 1 *vs* H_A_: *Δ* ≠ 1).

Then, the required total number of patients can be calculated as $\frac{d}{Probablity \left( of an event \right)}.$ A previous similar study conducted in Ghana showed that survival function (s (t)) = 0.16, thus we used$s\left( t \right)=0.16$. We also know that$\Delta=-\log\left( s\left( t \right) \right)= \log\left( 0.16 \right)=1.832$.

To obtain the total sample size required to yield a power of 90%, a hazard ratio of 1.832 and 5% two-sided level of significance, the required number of events is:

$d=\left( Z_{\frac{\alpha}{2}}+Z_{\beta} \right)^{2}\frac{4}{{In\Delta}^{2}}$=$\left( 1.96+1.282 \right)^{2}\frac{4}{{In(1.832)}^{2}} =115.$

Z_α/2_ = 1.96 for 95% confidence and Z_β =_ probability of committing type II error to give power of 90%.

Accordingly, $n=\frac{d}{Probablity (of an event)}=\frac{115}{0.84}=137.$

Adjusting for a loss of 20%. $n_{adj}=\frac{n}{1-loss}=\frac{137}{1-0.2}\approx172$.

The required sample size was 172. However, all colorectal cancer patients treated in TASH from 2012 to 2016 and who fulfil the inclusion criteria were included in the study. Hence the sample used for this study was 422.

**Table 1:**

Family history - of (any) cancer or of only CRC?

**Response:** Thank you for pointing this out. Amended as Family history of CRC.

**Table 2, Table 4 and Table 5**

Size and location of the tumor

Should be only Size of the tumor (no info about location, relevant data about location are under „Primary tumor site“).

**Response:** As requested, the correction has been made throughout the manuscript.

**Section: Result of multivariable Cox regression model**

Unify the style of writing of capital letters in list of variables over the whole section. In this list is again idiom „size and location of tumor“– should be only „size of tumor“.

**Response:** Thank you. We have made a uniform style of writing capital letters.

**Section: Discussion**

Line 323: unify style of writing capital letters for variables

**Response:** The suggested correction has been made.
